# Barriers and facilitators to women’s access to sexual and reproductive health services in rural Australia: a systematic review

**DOI:** 10.1186/s12913-024-11710-9

**Published:** 2024-10-11

**Authors:** Sarah M Wood, Laura Alston, Anna Chapman, Jacinta Lenehan, Vincent L Versace

**Affiliations:** 1 Deakin Rural Health, Deakin University, School of Medicine, Faculty of Health, Warrnambool Campus, PO Box 423, Warrnambool, VIC 3280 Australia; 2 Centre for Australian Research into Access, Deakin University, Warrnambool, VIC Australia; 3 Research Unit, Colac Area Health, Colac, VIC Australia; 4 Institute for Health Transformation, Centre for Quality and Patient Safety Research, School of Nursing and Midwifery, Faculty of Health, Deakin University, Geelong, VIC Australia; 5 Women’s Health and Wellbeing Barwon South West, Warrnambool, VIC Australia

**Keywords:** Sexual and reproductive health, Health service, Access, Systematic review, Delivery of healthcare, Health equity, Rural health, Barriers and facilitators, Women’s health

## Abstract

**Background:**

Accessing sexual and reproductive health (SRH) services in rural Australia presents complex challenges that negatively impact women’s health and exacerbate health inequities across the life course. This systematic review synthesises evidence on the barriers and facilitators to women’s access to SRH services in rural Australia, considering both supply and demand dimensions.

**Methods:**

We systematically searched peer-reviewed literature published between 2013 and 2023. Search terms were derived from three major topics: (1) women living in rural Australia; (2) spatial or aspatial access to SRH services; and (3) barriers or facilitators. We adopted the “best fit” approach to framework synthesis using the patient-centred access to healthcare model.

**Results:**

Database searches retrieved 1,024 unique records, with 50 studies meeting the inclusion criteria. Most studies analysed access to primary care services (*n* = 29; 58%), followed by hospital services (*n* = 14; 28%), health promotion and prevention (*n* = 5; 10%), and specialist care (*n* = 2; 4%). The type of care accessed was mostly maternity care (*n* = 21; 42%), followed by abortion services (*n* = 11; 22%), screening and testing (*n* = 8; 16%), other women’s health services (*n* = 6; 12%), and family planning (*n* = 4; 8%). There were numerous barriers and facilitators in access from supply and demand dimensions. Supply barriers included fragmented healthcare pathways, negative provider attitudes, limited availability of services and providers, and high costs. Demand barriers encompassed limited awareness, travel challenges, and financial burdens. Supply facilitators included health system improvements, inclusive practices, enhanced local services, and patient-centred care. Demand facilitators involved knowledge and awareness, care preferences, and telehealth accessibility.

**Conclusion:**

This review highlights the urgent need for targeted interventions to address SRH service access disparities in rural Australia. Understanding the barriers and facilitators women face in accessing SRH services within the rural context is necessary to develop comprehensive healthcare policies and interventions informed by a nuanced understanding of rural women’s diverse needs.

**Supplementary Information:**

The online version contains supplementary material available at 10.1186/s12913-024-11710-9.

## Background

Access to healthcare is a multifaceted indicator of healthcare system performance and equitable care provision [1]. Adequate access benefits individual health and positively impacts population health, while poor access contributes to health inequities and increases the disease burden [2]. Universal access to sexual and reproductive health (SRH) services has been a long-standing global priority, as barriers disproportionately impact women and contribute to health inequities across the life course [3–5]. Essential SRH services in primary care include family planning, maternity care, infertility treatment, abortion-related care, and the prevention, detection and treatment of sexually transmitted infections (STIs) [6]. The implications of sexual and reproductive ill-health extend beyond the disease burden, affecting the social and economic well-being of individuals, families, communities, and society [7, 8]. Access to SRH services is crucial for improving maternal health, reducing child mortality, and preventing communicable diseases [5]. Despite Australia’s reputation for advanced healthcare infrastructure, complex barriers and inequities persist in accessing SRH services in rural, regional, and remote (hereafter rural) areas.

**Fig. 1:**
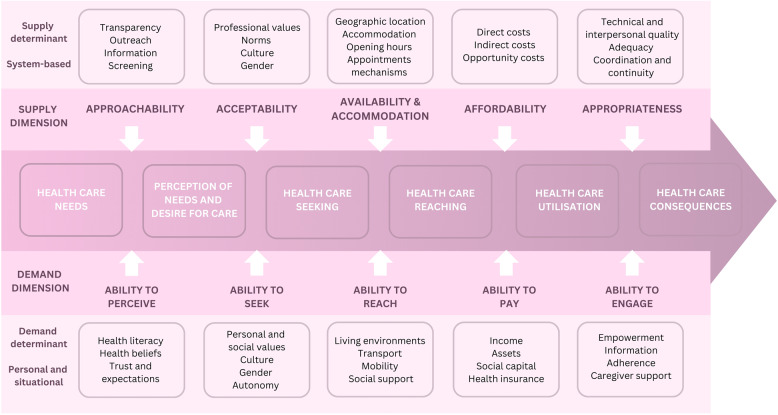
Patient-centred access to healthcare dimensions and determinants. (Adapted from Levesque et al. 2013 conceptual framework) [12]

Access is a complex concept, defined in spatial and aspatial terms [1]. Aspatial access relates to the non-geographic factors affecting access to services (e.g., affordability, acceptability), and spatial access relates to geographic factors affecting access (e.g., availability and accessibility) [9]. There are numerous models and frameworks for defining access to healthcare. Penchansky and Thomas’ framework is a commonly cited model that conceptualises access as the fit between the needs of patients and the healthcare system’s capacity [10, 11]. Levesque et al.’s [12] Conceptual Framework of Access to Healthcare (Fig. 1) presents a multidimensional view of healthcare access by considering both the supply and demand dimensions. The supply dimensions consider access in the context of health systems, such as the approachability, acceptability, availability and accommodation, affordability, and appropriateness of the healthcare supplied. Correspondingly, the demand dimensions focus on the abilities of individuals to interact with these dimensions of access, including their ability to perceive the need for healthcare, seek healthcare, reach healthcare, pay for healthcare, and engage in healthcare. These dimensions are interrelated constructs and often influence one another, depending on local health systems and patients’ characteristics [12]. The interaction between these supply and demand dimensions is influenced by various determinants, including individual, structural, and systemic factors. Considering access from both the health system (supply) and the patient’s perspective (demand) enables the development of strategies addressing service availability and utilisation disparities. Levesque’s et al.’s framework has previously been applied in studies to explore the dimensions of women’s access to SRH services, such as antenatal and maternity care [13, 14], telemedicine abortion [15], and contraceptives [16].

Australia’s inequitable distribution of health services is well documented, and issues associated with access are often influenced by Australia’s vast landmass, challenging geographic environments, and sparsely distributed populations in rural areas [9]. Most of Australia’s population lives in major cities, with only 28% living in rural and remote areas [17]. Similar to Canada and the United States, rural residents in Australia fare worse on most health and disadvantage indicators [18–20]. As remoteness increases, so do deaths that potentially could be avoided by addressing modifiable risk factors and preventative screening and treatment [19]. Geographic remoteness also introduces unique challenges in addressing disparities in access to SRH services [21]. For example, women living in rural Australia have higher fertility and maternal mortality rates than their urban counterparts, yet the inequitable distribution of maternity services has been a persistent challenge [22–25]. Existing literature has outlined barriers faced by rural women accessing SRH services, including greater travel distance from healthcare facilities [26], limited transport options, restricted service operating hours, service affordability, medical staff with limited SRH training, difficulties achieving confidentiality, and the risk of stigmatisation and discrimination [21, 27]. These challenges can lead to delayed or inadequate healthcare, which can have serious implications for the health and well-being of rural women.

Previous reviews have focused on specific types of SRH services, such as abortion services [28], but there is a significant gap in understanding the supply and demand dimensions influencing women’s access to the full spectrum of SRH services in rural Australia. Addressing this gap is essential for tackling inequities and shaping effective policy and intervention strategies. This systematic review, therefore, aims to identify the barriers and facilitators to women’s access to SRH services in rural Australian healthcare settings.

## Methods

This systematic review followed the Preferred Reporting Items for Systematic Reviews and Meta-Analyses (PRISMA) statement [29]. The completed PRISMA checklist is available in the supplementary materials (see Supplementary file 1). A protocol for this review was developed in advance and registered in PROSPERO (CRD42023482554).

### Eligibility criteria

The PICOS mnemonic was used to frame the inclusion and exclusion criteria (Table 1). *Population* included young women (15 to 24 years), adult women (25–44 years), and older women (65 to 74 years) [30] living in regional, rural, and remote Australia, defined by a geographical classification, such as the Modified Monash Model (MMM2-7) [31], Australian Statistical Geography Standard-Remoteness Areas (ASGS-RA1-4) [32] or otherwise defined by the authors.

**Table 1:** PICOS inclusion and exclusion criteria

Criteria	Inclusion	Exclusion
**P (Population)**	• Women living in regional, rural, or remote Australia aged 15–74	• Women living in metropolitan areas or outside of Australia
**I (Intervention)**	• Spatial or aspatial access to sexual and reproductive health services (e.g., maternity care, abortion services, contraception, fertility - IVF)	• Not focused on examining spatial or aspatial access. • Does not include sexual or reproductive health services
**C (Comparison)**	• none	
**O (Outcomes)**	• Barriers or facilitators for accessing sexual and reproductive health services	• Does not examine barriers or facilitators for accessing sexual and reproductive health services
**S (Study design)**	• Primary research; quantitative, qualitative, and mixed-methods	• Study protocols, editorials, conference abstracts, grey literature
**Time period**	• 1 January 2013 to 15 November 2023	• Publication dates outside of 1 January 2013 and 15 November 2023

*Intervention* included the examination of spatial and aspatial access to an SRH service Following the World Health Organization life course approach [33], SRH services must cover access to contraception, menstrual health and menopause, fertility and infertility care, maternal and perinatal health, prevention and treatment of STIs, and education. SRH services were classified and presented according to the Australian Institute of Health and Welfare (AIHW) definitions to reflect the various components of the Australian healthcare system (Table 2). *Outcomes* included the barriers and/or facilitators to women’s access to SRH services. Barriers and/or facilitators could be reported either by women or healthcare providers. Literature published in English in a peer-reviewed journal between 1 January 2013 and 15 November 2023 was included. The past ten years were chosen to correspond with the regulated and subsidised availability of medical abortion in Australia [34]. Primary research studies that utilised a quantitative, qualitative, or mixed-methods design were considered eligible for inclusion. Study protocols, editorials, conference abstracts, and grey literature were excluded from this review.

**Table 2:** Types of sexual and reproductive health services in Australia

Health services	Definition	Example of services
Health promotion and prevention	Improving health and preventing ill health	• Immunisation and vaccination • Cervical screening • STI screening • Disease prevention programs
Primary healthcare	First contact with the health system	• General practitioner for services such as contraception counselling, intrauterine device insertion, medical abortion prescription • Pharmacy for services such as dispensing of emergency contraception and medical abortion medicine • Community health and family planning
Specialist care	Provides services for those with specific or complex conditions or issues	• Referred medical specialist services, such as gynaecology, obstetrics, and fertility services • Diagnostic services
Hospitals	Services provided to admitted and non-admitted patients	• Inpatient • Outpatient clinics • Emergency department care

Source: Adapted from AIHW [35] and Wood et al. [1]

### Information sources

A literature search was conducted on 15 November 2023, using six electronic databases: EMBASE (Elsevier), CINAHL (EBSCOhost), Health Policy Reference Center (EBSCOhost), Health Source: Nursing/Academic Edition (EBSCOhost), Global Health (EBSCOhost), and MEDLINE Complete (EBSCOhost).

### Search strategy

A comprehensive list of search terms was developed from a preliminary search of MEDLINE and CINAHL databases and a review of relevant literature, such as SRH systematic reviews. The keywords in the titles and abstracts of relevant articles and the Medical Subject Headings (MeSH) terms used to index articles were utilised to develop the full search strategy. A combination of search terms related to the following concepts: [1] women living in rural, regional, or remote Australia; [2] spatial or aspatial access to SRH services; and [3] barriers or facilitators to access. A supplementary file outlines the complete search strategies (see Supplementary file 2). A librarian with expertise in developing search strategies for health databases reviewed the searches. The search strategy, including all identified keywords and index terms, was adapted for each database.

### Screening and selection

All identified citations were collated and uploaded into Endnote (Version 20.2.1, Clarivate, Philadelphia, PA). Citations were then imported into Covidence (Veritas Health Innovation, Melbourne, Australia), and duplicates were removed. Titles and abstracts were dual-screened in Covidence by three independent reviewers (SW, LA, AC) using the prespecified eligibility criteria. Potentially relevant studies were retrieved as full texts and dual-assessed in detail against the inclusion criteria by three independent reviewers (SW, LA, AC). Reasons for exclusion at the full-text stage were recorded. Reference lists of included studies were screened for additional studies. Any reviewer disagreements during the selection process were resolved through discussion with the third reviewer.

### Data extraction

Data were extracted from eligible studies by SW using Excel and tabulated with the following headings: author, year, health service setting, health discipline, context, geographic location according to MMM or ASGS-RA, population characteristics, study objective, study design, barriers, facilitators, summary of findings, study limitations, and implications. Ten per cent of the extracted data were cross-checked by another reviewer (LA). Any reviewer disagreements were resolved through discussion or with an additional reviewer.

### Quality assessment

Quality assessment of included studies was conducted using the relevant Joanna Briggs Institute (JBI) critical appraisal tools for the appropriate study design (e.g., analytical cross-sectional studies, qualitative studies, cohort studies) [36]. Two quality appraisal tools were used for studies applying mixed methods (e.g., JBI qualitative and cohort checklists). SW completed the quality appraisal, and to ensure accuracy, another reviewer (LA) verified ten per cent of the appraisals. Any discrepancies between the reviewers were resolved through consultation with a third reviewer.

### Data synthesis and patient-centred access model analysis

Study characteristics (e.g., barriers or facilitators, SRH service) were tabulated and synthesised narratively to describe the available evidence. The “best fit” approach to framework synthesis outlined by Carroll et al. [37] was undertaken using Levesque et al.’s [12] patient-centred access to healthcare model. This method has also been used in previous systematic reviews [13]. The a priori framework provided the relevant themes (access dimensions) to enable a deductive approach to coding and categorising barriers and facilitators from the studies identified for this review. Barriers and facilitators were extracted according to how they were reported in the study (e.g., barrier or facilitator) and summarised based on the type of SRH service and whether they were from the provider’s or patient’s perspective. The barriers and facilitators were then coded and categorised according to the supply or demand dimension of Levesque et al.’s model. Barriers and facilitators relating to the supply of healthcare were analysed according to the supply dimensions: approachability, acceptability, availability and accommodation, affordability, and appropriateness. Barriers and facilitators relating to the abilities of individuals to interact with healthcare were analysed according to the demand dimensions: ability to perceive, ability to seek, ability to reach, ability to pay, and ability to engage. Studies that included perspectives from both the provider and patient were analysed in the appropriate dimension of the framework. In synthesising the data against the Levesque et al. framework, the research team acknowledges the varied disciplinary backgrounds and perspectives that can influence data interpretation and synthesis. The authors come from backgrounds in rural health (SW, LA, AC, VLV), women’s health (SW, JL, VLV), and health geography (SW, VLV). The first author (SW) conducted data synthesis, and there were ongoing discussions with the other authors during this process to review the coded data against the themes. The patient-centred access model provided the a priori framework to guide the “best fit” approach to framework synthesis and mitigate bias.

## Results

**Fig. 2:**
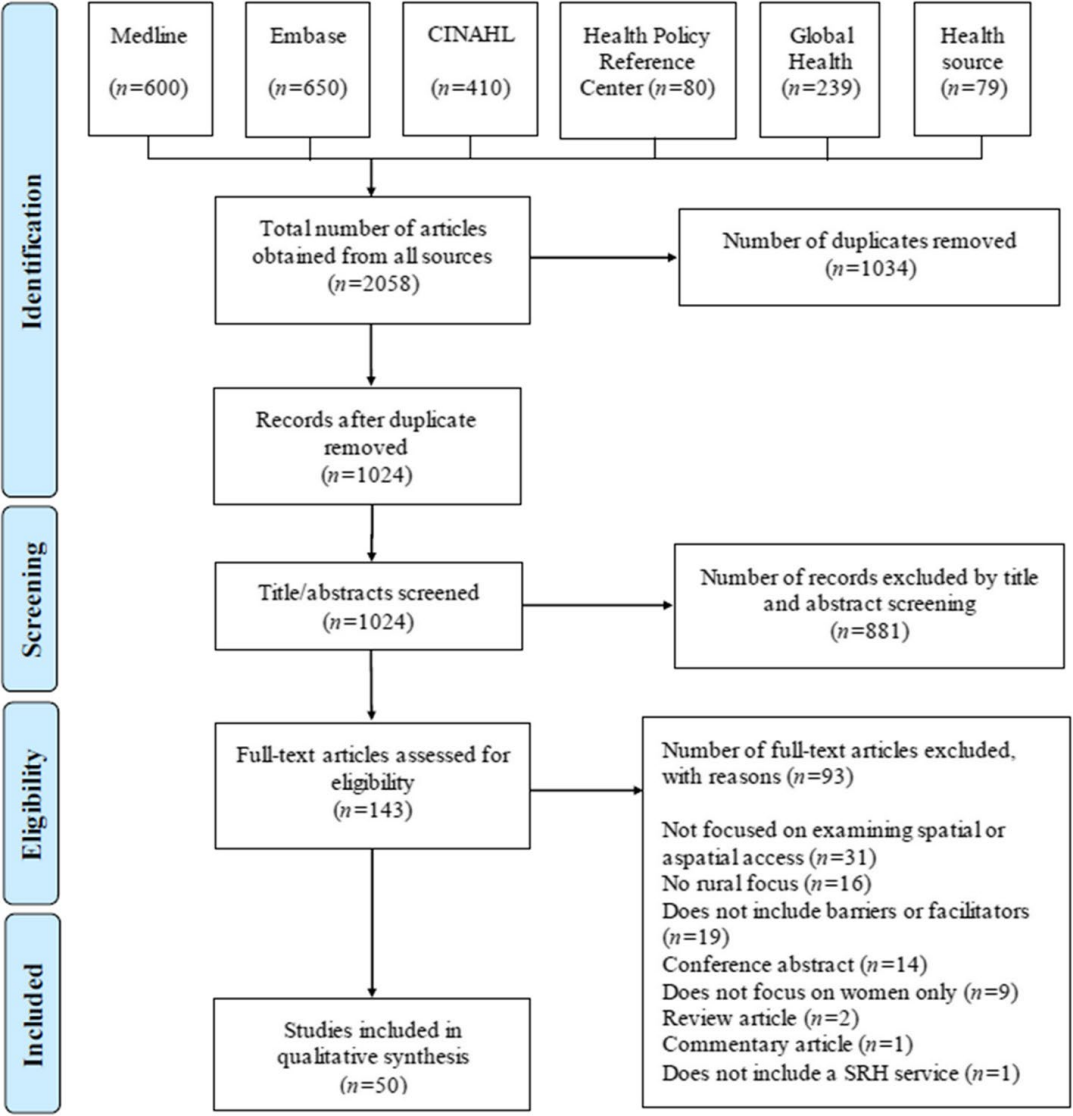
PRISMA flow diagram of screening process

The search retrieved 1,024 unique records that were screened for inclusion based on their title and abstract with 50 articles meeting the inclusion criteria. Reasons for exclusion at the full-text phase are described in the PRISMA diagram (Fig. 2).

### Characteristics of selected studies

The characteristics of the 50 included studies are presented in Table 3. Most studies analysed women’s access to primary care services (*n* = 29; 58%), followed by hospital services (*n* = 14; 28%), health promotion and prevention (*n* = 5; 10%), and specialist care (*n* = 2; 4%). Over half (*n* = 28) of the studies examined barriers and facilitators from the women’s perspective, just under half (*n* = 25) were from the provider or clinician’s perspective, and four examined barriers and facilitators from both perspectives. Of the 50 studies, most studies employed a qualitative approach to data collection (*n* = 33; 66%), followed by quantitative (*n* = 11;22%), and mixed-methods (*n* = 6; 12%).

**Table 3:** Summary table of included articles (*n* = 50)

First author, citation	Year	Type of care	Perspective	Context	Population characteristics	Location	Data collection approach
***Primary care services (n*** ***= 29)***							
Campbell [22]	2014	Maternity care	Healthcare provider	Factors influencing rural GP-obstetric practice	Rural GP (*n =* 22)	Rural VIC	Qualitative
Cashman [23]	2021	Sexual health service	Healthcare consumer	Experiences accessing MToP	Women who accessed MToP (*n =* 11)	Outer regional QLD	Qualitative
Dawson [25]	2017	General practice	Healthcare provider	Provision and referral of MToP by GPs	GPs (*n =* 32)	Metro to very remote NSW	Qualitative
De Moel-Mandel [26]	2020	Primary health services	Healthcare provider	Nurse-led model for early MToP provision	Medical and other health professionals (*n =* 24)	Regional and rural VIC	Qualitative
Doran [11]	2014	Women’s health centre	Healthcare provider	Experiences accessing SToP	Staff from community-based non-government Women’s Health Centres (*n* = 7) Rural women who had an abortion in the last 15 years (*n =* 13)	Rural and regional NSW	Qualitative
Doran [28]	2016	Women’s health centre	Healthcare consumer	Factors that impact the experiences of accessing a SToP	Rural women who had an abortion in the last 15 years (*n =* 13)	Rural and regional NSW	Qualitative
Dutton [29]	2020	Community health	Healthcare consumer	Uptake and acceptability of HPV self-sampling model	Aboriginal women aged 25–69 years of age (*n =* 215)	Rural and remote NSW	Quantitative
Foo [30]	2021	General practice	Healthcare provider	Experiences and attitudes of GPs towards NCSP self-sampling option	Rural GPs (*n =* 12)	Rural and regional NSW	Qualitative
Grant [32]	2019	General practice	Healthcare consumer	Experiences of routine sexual healthcare	Bisexual rural women (*n =* 15)	Regional and rural TAS	Qualitative
Gudka [33]	2014	Pharmacy	Healthcare consumer	Self-reported risk factors for chlamydia in pharmacy-based EC consumers	Women accessing emergency contraception (*n =* 113)	Metro, rural, regional, remote WA	Quantitative
Hulme-Chambers [37]	2018	Primary healthcare service	Healthcare provider	Factors that enabled and challenged a decentralisation effort to increase rural MToP service provision	Training providers and participants (*n =* 19)	Rural VIC	Qualitative
Hulme-Chambers [38]	2018	Primary healthcare service	Healthcare consumer	Experiences of women obtaining a MToP	Rural women who accessed MToP (*n =* 18)	Rural VIC	Qualitative
Ireland [39]	2020	Telehealth	Healthcare consumer	Understand women’s access to telemedicine abortion	Rural women who accessed a telehealth MToP (*n =* 11)	Outer regional to very remote NSW, NT, TAS	Qualitative
Josif [40]	2014	Maternity care	Healthcare provider	Experiences of a new model of maternity care for remote dwelling Aboriginal women	Health professionals, Aboriginal health workers, Department of Health staff, and Aboriginal women who gave birth (*n =* 66)	Remote NT	Qualitative
Keough [41]	2019	General practice	Healthcare provider	GP knowledge and practice regarding unintended pregnancy	GPs (*n =* 28)	Regional VIC	Mixed-methods
Kruss [43]	2014	Family planning services	Healthcare provider	Barriers to accessing family planning services	Professionals connected to family planning services (*n =* 11)	Regional and rural VIC	Qualitative
Lorch [47]	2015	General practice	Healthcare provider	PN chlamydia testing	Practice nurses (*n* = 23)	Rural NSW, QLD, SA, VIC	Qualitative
Makleff [48]	2023	Abortion services	Healthcare consumer	Impact of abortion stigma on quality of care	Women seeking abortion (*n =* 24)	Regional AUS	Qualitative
Malatzky [49]	2022	General practice	Healthcare provider	Challenges encountered by rural sexual and reproductive health practitioners	Sexual and reproductive health practitioners (*n =* 15)	Rural VIC	Qualitative
Moel-Mandel [50]	2019	General practice	Healthcare provider	Enablers and barriers to MToP provision	Rural GP (*n =* 39) and primary healthcare nurse (*n =* 30)	Regional and rural VIC	Quantitative
Munns [51]	2021	Community health	Healthcare provider	Experiences from a community midwifery-led antenatal program	Community midwives, child health nurses, program managers, a liaison officer, doctors and community agency staff (*n =* 19)	Remote WA	Quantitative
Noonan [52]	2022	General practice	Healthcare consumer	Experiences of managing unintended pregnancy in a rural area	Rural women who experienced an unintended pregnancy in the past five years (*n =* 20)	Rural NSW	Qualitative
Noonan [53]	2023	General practice	Healthcare consumer	Experiences locating services for unintended pregnancy in a rural health system	Rural women who experienced an unintended pregnancy in the past five years (*n =* 20)	Rural NSW	Qualitative
Roxburgh [55]	2021	General practice	Healthcare consumer	Satisfaction with GP obstetrician-led maternity care	Rural pregnant women ≥ 24 weeks gestation (*n =* 155)	Regional, rural, and remote WA	Mixed-methods
Rumbold [56]	2015	Maternity care	Healthcare provider	Barriers to providing fetal anomaly screening to Aboriginal women	Professionals that care for pregnant Aboriginal women (*n =* 59)	Remote NT	Qualitative
Sivertsen [61]	2021	Community health	Healthcare consumer	Knowledge and experiences of women’s health community services	Women’s health service clients (*n =* 13)	Regional, rural, and remote NSW	Qualitative
Subasinghe [62]	2021	General practice	Healthcare consumer	Provision of MToP from PBS claims	Women aged 15–54 years	Inner regional to very remote AUS	Quantitative
Telford [64]	2022	General practice Maternity care	Healthcare provider	GP obstetrician perspectives of practising in the region	GP obstetricians (*n =* 11)	Regional and rural NSW	Mixed-methods
Zadoroznyj [67]	2013	Community health	Healthcare provider	Pharmacy nurse providers of community-based post-birth care	Pharmacy nurses (*n =* 19) and GPs (*n* = 6)	Regional and rural QLD	Qualitative
***Hospital services (n*** ***= 14)***							
Bar-Zeev [20]	2014	Maternity care	Healthcare provider	Quality of antenatal care	Clinicians (*n =* 27) Aboriginal women (*n =* 412)	Remote NT	Mixed-methods
Brown [21]	2016	Maternity care	Healthcare consumer	Experiences of accessing standard hospital care for birth	Aboriginal and Torres Strait Islander women (*n =* 14)	Rural and remote SA	Qualitative
Doig [27]	2021	Maternity care	Healthcare provider	Antenatal point-of-care ultrasound services	Medical professionals (*n* = 26)	Regional, rural, and remote SA	Quantitative
Hennegan [34]	2014	Maternity care	Healthcare consumer	Impact of remoteness and rurality on experiences of care	Women (*n =* 6995)	Inner regional to very remote QLD	Quantitative
Hoang [35]	2013	Maternity care	Healthcare consumer	Rural women’s needs in maternity care	Rural women (*n =* 210)	Rural TAS	Mixed-methods
Hoang [36]	2014	Maternity care	Healthcare consumer	Impact of the lack of maternity services	Rural women (*n =* 210)	Rural TAS	Mixed-methods
Kruske [42]	2016	Maternity care	Service provider	PMU existence in rural and remote Australia	PMUs maternity services (*n =* 17)	Outer regional to very remote AUS	Quantitative
Longman [46]	2017	Maternity care	Healthcare provider	Barriers to operationalising national policy for maternity services in rural and remote Australia	Health service leaders and managers, policymakers, service providers, clinicians and consumers (*n =* 141)	Rural and remote NSW, NT, QLD, WA	Qualitative
Rolfe [54]	2017	Maternity care	Healthcare provider	Distribution of maternity services across rural and remote Australia	Maternity services located in rural and remote Australia (*n* = 259)	Inner regional to very remote AUS	Quantitative
Russell [57]	2021	Maternity care	Healthcare consumer	Factors that influence rural women’s choices in maternity care	Women who birthed in rural Vic (*n =* 10)	Rural VIC	Qualitative
Seear [59]	2021	Maternity care	Healthcare consumer	Experiences of antenatal care	Aboriginal women accessing antenatal care (*n =* 124)	Regional, rural, and remote WA	Qualitative
Shackleton [60]	2023	Maternity care	Healthcare consumer	Experiences of travelling long distances and/or relocating to give birth	Rural and remote mothers (*n =* 9)	Rural and remote WA	Qualitative
Sweet [63]	2015	Maternity care	Healthcare consumer	Map and analyse the change in maternity services over the past 20 years	Birth location for rural South Australian women from 1991 to 2010	Inner regional to very remote SA	Quantitative
Wong Shee [66]	2021	Maternity care	Healthcare consumer	Teenage women’s experiences of engaging in pregnancy care	Pregnant women aged < 19 years (*n =* 16)	Regional and rural VIC	Qualitative
***Health promotion and prevention (n*** ***= 5)***							
Christie [24]	2023	Breast cancer screening	Healthcare consumer	Culturally safer pathways that improve participation in screening and treatment	Indigenous women (*n =* 21)	Regional and rural NSW	Qualitative
Gosbell [31]	2023	National Cervical Screening Program	Healthcare consumer	Awareness and attitudes towards the revised NCSP	Rural women (*n =* 309)	Rural NSW	Quantitative
Lafferty [44]	2021	STI testing	Healthcare provider	Scaling up point-of-care testing	Healthcare workers from remote health services (*n =* 15)	Remote and very remote SA, TAS, QLD, WA	Qualitative
Lansbury [45]	2023	Menstrual health	Healthcare consumer Healthcare provider	Experiences of menstrual health for Indigenous girls in a remote area	Indigenous girls (*n =* 72) Health professionals (*n =* 15)	Remote and very remote QLD	Qualitative
Wagg [65]	2020	STI testing	Healthcare consumer	Understanding of chlamydia and factors that may prevent or delay testing	Young women aged 18–30 years (*n =* 11)	Regional and rural VIC	Qualitative
***Specialist care (n*** ***= 2)***							
Arnold [19]	2021	Gynaecology oncology	Service provider	Telehealth satisfaction	Patients who accessed the service (*n =* 53)	Outer regional QLD	Quantitative
Sassano [58]	2023	Reproductive services	Service provider	Ethical implications of maldistribution of ART services in rural areas	Women living in regional areas accessing ART (*n* = 12)	Regional, rural, and remote NSW, QLD, TAS, VIC	Qualitative

*ART* assisted reproductive technology, *AUS* Australia, *EC* emergency contraception, *GP* general practitioner, *HPV* human papillomavirus, *MToP* medical termination of pregnancy, *NCSP* National Cervical Screening Program, *NSW* New South Wales, *NT* Northern Territory, *PBS* Pharmaceutical Benefits Scheme, *PMU* primary maternity units, *PN* practice nurses, *QLD* Queensland, *SA* South Australia, *STI* sexually transmitted infection, *SToP* surgical termination of pregnancy, *TAS* Tasmania, *VIC* Victoria, *WA* Western Australia

The highest proportion of studies were conducted in New South Wales (NSW) (*n* = 14; 23%), followed by Victoria (VIC) (*n* = 13; 22%). The geographic classifications used to define rurality or remoteness areas varied across the studies, including the ASGS-RA (*n* = 10), Australian Statistical Geography Classification Remoteness Areas (ASGC-RA) (*n* = 3), Accessibility/Remoteness Index of Australia (ARIA, ARIA+) (*n* = 3), and MMM (*n* = 1) (Supplementary File 4). Thirty-three studies did not use a geographic classification to define the location.

### Service setting

The highest proportion of studies were focused on maternity care settings (*n* = 21; 42%), followed by abortion services (*n* = 11; 22%), screening and testing (e.g., STI, cervical, breast screening) (*n* = 8; 16%), other women’s health services (*n* = 6; 12%), and family planning (*n* = 4; 8%). Other women’s health services encompassed gynaecology, sexual healthcare, reproductive services, and experience accessing a community-based women’s health clinic. Studies investigating access to maternity care focused on various aspects of care, including antenatal and postnatal services in regional, rural, and remote Australia. Six studies were focused on Aboriginal and Torres Strait Islander women’s experience of maternity care. Studies examining access to abortion services were primarily focused on rural and regional access to medical termination of pregnancy (MToP) (*n* = 8), as opposed to surgical termination of pregnancy (SToP) (*n* = 2), and one study examined both. Studies that focused on screening and testing included sexually transmissible infections (STI) (*n* = 4), cervical screening programs (*n* = 3), including self-sampling (*n* = 2), and breast cancer screening (*n* = 1). Family planning predominantly focused on rural and regional women’s experiences of unintended pregnancy and accessing family planning services, such as options counselling, emergency contraception, abortion services, or antenatal care.

### Barriers and facilitators

Key barriers and facilitators are presented under the five dimensions of the patient-centred access to healthcare model (Fig. 1) [12]. Barriers and facilitators are presented for both the supply and demand dimensions of the model, including approachability (ability to perceive), acceptability (ability to seek), availability and accommodation (ability to reach), affordability (ability to pay), and appropriateness (ability to engage). It is important to note that some of the supply and demand factors complement each other.

#### Approachability and ability to perceive

Approachability represents the capacity of a health system to provide services so that women with health needs can identify and reach them [12]. Health services can make themselves known to women from different social, cultural, or geographical groups through transparency, providing information (e.g., about available treatments), and outreach activities. Individual factors such as health literacy, knowledge and beliefs about health determine the ability to perceive healthcare needs. The dimensions of approachability and ability to perceive were explored in 28 studies across primary care (*n* = 10), hospital (*n* = 10), and health promotion and prevention (*n* = 8) settings.

##### Supply barriers

Three main barriers were identified for approachability: insufficient information, fragmented healthcare pathways, and limited internet access. Insufficient information primarily related to gaps in the information provided by GPs around maternity care providers [38] and abortion options [15, 39]. Another study reported that women lacked information on available local services and support [40]. Studies reported gaps in provider knowledge and awareness around abortion, contributing to insufficient information [41–44]. Fragmented healthcare pathways were noted in two studies [45, 46], particularly concerning access to local maternity care providers and options for unintended pregnancy. Under the dimension of approachability, fragmented pathways refer to the lack of transparency and visibility of services, making it difficult for women to identify and access the care they need. The healthcare pathways for unintended pregnancy were described as unclear and disjointed compared to those for antenatal care [45]. Providers also noted the lack of referral pathways, particularly to post-natal care [47]. Limited internet access was described as a barrier inhibiting individuals from obtaining information about various healthcare services, contributing to restricted awareness and choice of providers [48].

##### Demand barriers

Three main barriers emerged related to women’s ability to perceive healthcare: low health literacy, limited awareness, and difficulties in navigating the health system. The barrier of low health literacy was predominantly related to cervical screening [49] and STI symptoms, testing, and treatment [50]. Limited awareness was identified for available information regarding local and financial support, such as the Patient Assisted Travel Scheme (PATS) for those required to relocate [46]. Difficulties in navigating the health system and coordinating services were reported in studies focused on unintended pregnancy [45] and maternity care [46]. For example, women reported difficulties in locating appropriate services, often relying on word of mouth or personal contacts to identify services [45].

##### Supply facilitators

Two main facilitators were identified for approachability: health system improvements and enhanced education and institutional approaches. Health system improvements pertained to changes to the health system, such as providing culturally accessible information and roles for Aboriginal Health Practitioners (AHP) to work with the community and coordinate access to screening services [51]. Additional improvements included direct referrals from GPs to services [52] and utilising midwifery group practice models for more efficient health system navigation for antenatal care [53]. The second facilitator was enhanced education and institutional approaches. This encompassed recommendations for comprehensive, culturally respectful, and accessible information about puberty and menstruation within the school health curriculum [54]. One study [55] suggested using a whole-of-institutional approach in healthcare settings (similar to whole-school approaches) to support change at multiple levels, and shifting institutional culture around abortion through de-stigmatising policies and protocols. Finally, improved dissemination of information regarding available relocation subsidies was recognised as a facilitator to alleviate the financial burden on women relocating for maternity care [46].

##### Demand facilitators

Three facilitators were identified for women’s ability to perceive healthcare: information access, knowledge and awareness, and culturally appropriate information. Information access was primarily related to internet access, as this was the most common way women accessed information, and ease of access to information determined the choice of provider [48]. Knowledge and awareness were key themes in several studies, predominantly focusing on awareness of services [56] and enhancing knowledge about symptoms and testing of STIs [50]. The final facilitator related to the ability to perceive was culturally appropriate information, such as suitable health promotional material for Aboriginal and Torres Strait Islander women and culturally inclusive education sessions for antenatal care [51, 57, 58].

#### Acceptability and ability to seek

Acceptability relates to cultural and social factors that affect a woman’s ability to accept or seek health services or aspects of them. For example, the gender or social group of the provider or the beliefs associated with systems of medicine may reduce the acceptability of seeking care [12]. The ability to seek care is related to the concepts of autonomy and the capacity to choose to seek care, knowledge about healthcare options, and individual rights that determine the intention to obtain healthcare. The dimensions of acceptability and ability to seek were explored in 30 studies across primary care (*n* = 15), hospital (*n* = 10), and health promotion and prevention (*n* = 5) settings.

##### Supply barriers

Three primary barriers were identified for acceptability: negative provider attitudes, low-quality care, and privacy concerns. Negative provider attitudes were frequently reported by women and included GPs’ unwillingness to refer for abortion services or denying care altogether, leaving women needing to locate willing providers themselves [15, 39, 52, 55, 59]. Provider resistance, stigma and religious objections were reported in studies focused on abortion or family planning services [41, 59, 60]. Additionally, negative provider attitudes were reported across studies providing other health services, including STI testing [50] and maternity care for Aboriginal women [61]. Low-quality care was a reported barrier in studies of abortion services, specifically the impact of abortion stigma on the quality of care in the context of healthcare interactions [55]. Privacy concerns were identified across multiple studies, including family planning [59], abortion services [15], and STI testing [62].

##### Demand barriers

Three barriers were identified for women’s ability to seek healthcare: lack of cultural safety, stigma, embarrassment and perceived judgment, and community context challenges. Lack of cultural safety primarily affected maternity care for Aboriginal and Torres Strait Islander women, whereby standard maternity care was not deemed culturally safe, with limited cross-cultural understanding of medical care [63]. The absence of inclusive services was a barrier to sexual health services [64]. Stigma, embarrassment, and perceived judgment from health professionals were barriers highlighted in a study involving young women regarding STI testing [50]. Community context challenges related to the inherent challenges of living in a small rural community [45, 59]. Some studies reported barriers, such as fear of stigmatisation and the lack of confidentiality in a small town, rural culture, and community ties [45], as well as limited local resources resulting in a lack of agency [46].

##### Supply facilitators

Three facilitators were identified for acceptability: inclusive and culturally sensitive practices, enhanced privacy with flexible service delivery, and improved health system practices. Culturally sensitive practices involved ensuring cultural safety and consulting women on their cultural needs around birth [63]. Inclusive practices identified in studies related to communication, the use of gender-neutral language, and the incorporation of visual signs of an inclusive environment [64]. Several facilitators pertaining to enhanced privacy with flexible service delivery were observed, including self-sampling for cervical screening tests and telehealth for MToP appointments [15, 65]. Self-cervical screening not only increased privacy but also empowered Aboriginal women to be in charge of women’s business [65]. Improved health system practices related to services, such as normalising family planning within the health system, including the right to make reproductive decisions regarding if, how and when to have children [59]. Additionally, the provision of high-quality abortion care with active assistance from healthcare providers in accessing such care [55].

##### Demand facilitators

The main facilitator for women’s ability to seek healthcare was related to care preferences. Birthing in the local community was reported to be a positive experience for women [66]. Services that offered greater discretion and privacy were reported as facilitators in several studies, particularly in rural areas [15, 48]. The preference for communication style regarding care was also noted as a facilitator [56, 67]. For example, some studies found that text messaging assisted in managing appointments and providing pertinent information to patients, particularly for younger age groups. Conversely, older patients were perceived to prefer receiving a letter in the post [56, 67].

#### Availability and accommodation and ability to reach

Availability and accommodation refer to the ability to access health services and healthcare providers, both physically and promptly. Availability means enough health resources to provide services, including the providers’ characteristics (e.g., presence of the health professional, qualification) and how services are delivered [12]. Factors such as a woman’s mobility, transportation access, employment flexibility, and knowledge about health services determine the ability to reach healthcare. The dimensions of availability and accommodation and ability to reach were examined in 38 studies across hospital (*n* = 19), primary care (*n* = 10), health promotion and prevention (*n* = 6), and specialist care (*n* = 3) settings.

##### Supply barriers

Three barriers were identified for availability and accommodation: poor local access, inadequate health service availability, and limited provider availability. Poor local access was related to the lack of and fragmented local services. For example, ultrasound or pathology services were not available at the same location as the care provider, requiring patients to travel [58, 61, 68]. Limited local aftercare and support were identified as a barrier to postnatal care [69, 70]. For instance, women who travel to another location to give birth were limited in local postnatal support once returning [69, 70]. Inadequate health service availability was attributed to women’s difficulty in obtaining appointments due to limited availability or long wait times [48, 52, 59, 66, 70, 71]. Women also reported delays in care or procedures due to long wait times for pre-appointment screenings, such as blood tests or ultrasounds [39, 48, 59]. Limited provider availability was particularly noted for female doctors, rural GPs, and AHPs [21, 59, 61, 70]. Studies also reported a lack of available providers for MToP appointments [70], pharmacists dispensing mifepristone and misoprostol [41], and a low number of rural practitioners willing to provide abortion or obstetric services [45]. Rural workforce challenges were a prominent barrier reported by providers [57, 58, 72–74]. Recruitment and retention of midwives, obstetricians, GP obstetricians, and paediatricians was reported to be one of the biggest threats to the sustainability of rural maternity services [73–75].

##### Demand barriers

Three barriers were identified that impacted a woman’s ability to reach healthcare: transportation issues, increased travel, and relocation for care. Transportation issues were particularly relevant for women without a driving licence or car, as they had to depend on public transportation or family and friends [67]. Increased travel was a commonly reported barrier across most studies, including long distances to access services [21, 39, 61, 66, 69, 71], travel-related inconveniences [40], and the distance to culturally appropriate care [51]. For example, one study [61] reported that Aboriginal women in remote communities had to travel long distances, including night-time travel by public bus for antenatal appointments, with only one available return bus service per day. Relocation for care was predominantly reported in studies focused on maternity care for Aboriginal and Torres Strait Islander women [61, 63].

##### Supply facilitators

Facilitators identified for availability and accommodation encompassed the enhancement of local service provision and capacity, and increased resource accessibility. Strategies to enhance local service provision and capacity involved increasing their capacity [21], optimising the use of shared maternity care and telehealth [46], MToP telehealth availability [15], and on-site ultrasound services [48]. Additional facilitators related to new services, such as reopening local services [46], rural birthing services [66], early postnatal supports [66], and an increased number of pharmacies stocking mifepristone and misoprostol [60]. Local access to an Indigenous midwife or AHP, as well as the provision of maternity home visits for Aboriginal communities was also identified [61, 63]. Finally, increased resource accessibility predominantly centred on menstrual health and access to sanitary products. Facilitators included providing free and discreet sanitary products and waste disposal facilities, availability of washing amenities and pain management in schools, and improving the availability of environmentally friendly, reusable products in rural locations [54].

##### Demand facilitators

One facilitator was identified for women’s ability to reach healthcare: time and travel efficiency. This was related to telehealth appointments [76] and human papillomavirus (HPV) self-screening [65], which reduced travel requirements and saved time.

#### Affordability and ability to pay

Affordability pertains to a woman’s economic ability to allocate resources and time to necessary health services. The direct costs of services, related expenses, and the potential loss of income influence it. Affordability can differ based on the type of service and relies on the ability to acquire the resources needed to cover care costs [12]. The ability to pay describes the capacity to generate economic resources through income for healthcare services without suffering catastrophic financial consequences, such as selling a home. Factors such as poverty, social isolation, or debt can restrict a woman’s ability to pay for necessary care. The dimensions of affordability and ability to pay were explored in 26 studies across hospital (*n* = 10), primary care (*n* = 7), health promotion and prevention (*n* = 6), and specialist care (*n* = 3) settings.

##### Supply barriers

Two primary barriers to affordability were identified: high direct costs and costs associated with essential products. The high direct costs identified across multiple studies included limited bulk-billing options [50, 56], the cost of health services [56], and the elevated costs of local providers [52]. One study [52] reported that the local provider was more expensive than travelling to a nearby town for the same service. Other studies reported cost barriers associated with emergency contraception and abortion services [39, 59]. The costs of essential sanitary products were also found to be high in rural areas, with local stores stocking low-quality options at expensive prices [54].

##### Demand barriers

Two barriers were found to hinder a woman’s ability to pay for healthcare: high travel-related costs and the financial burden of indirect costs. High travel-related costs were reported in four studies for women who were required to travel for care [40, 51, 56, 70]. Several studies identified the financial burden of indirect costs [15, 21, 39, 48, 59, 60]. Examples of indirect costs included transportation, accommodation, childcare, and loss of wages. Significant financial burdens were reported for women who needed to relocate for maternity care [46, 63]. For example, one study [46] reported the lack of financial support for partners who also relocated and incurred accommodation expenses while the mother was in the hospital.

##### Supply facilitators

Two facilitators emerged for affordability, including cost reduction strategies, and policy and incentive support. Cost reduction strategies involved the increased use of telehealth services due to lower associated costs [76]. Policy and incentive support included paid and subsidised local abortion service provisions to reduce travel [39], and nationally consistent pricing on sanitary products [54].

##### Demand facilitators

Service affordability was identified as a facilitator for women’s ability to pay for healthcare. Improving access and availability of MToP appointments and medication in rural areas was found to be much more affordable than accessing SToP [15]. Increased use of HPV self-sampling was another facilitator reported in studies, as this was often a no-cost service [65].

#### Appropriateness and ability to engage

Appropriateness refers to the overall clinical benefit for the woman and whether the expected health benefits (e.g., improved quality of life) outweigh the potential negative consequences (e.g., time, cost). Appropriateness also includes adequacy, which pertains to the quality of the type and model of services provided and their continuity [12]. The ability to engage relates to the woman’s participation in decision-making and treatment decisions, which is strongly influenced by the capacity to participate. The dimensions of appropriateness and ability to engage were explored in 28 studies across hospital (*n* = 13), primary care (*n* = 9), health promotion and prevention (*n* = 3), and specialist care (*n* = 3) settings.

##### Supply barriers

Two barriers were found for appropriateness: limitations in telehealth accessibility and care discontinuities. The limitations in telehealth accessibility were linked to language barriers for non-English speaking women and the absence of visual cues for the visually impaired [76]. Care discontinuities were documented in several studies relating to maternity care, particularly when women had to travel for care and had limited postnatal care options [45, 46, 69]. In one study, women reported concerns about care discontinuities in the context of assisted reproductive services due to facility shortages and restricted service provision [77].

##### Demand barriers

Two barriers were also identified for women’s ability to engage in healthcare, including a lack of support systems and psychological effects. Lack of support systems pertained to women who were required to relocate to give birth. Women who relocated experienced disconnection and distress from leaving family support and also reported inadequate caregiver support for existing children [63]. Another study reported that travelling to access abortion services meant leaving crucial support systems [59]. Insufficient childcare options to enable travel for care were also noted within lack of support systems [21]. Finally, the psychological effects stemming from access burdens and distress when trying to locate services was another identified barrier [59].

##### Supply facilitators

Two facilitators were identified for appropriateness, including telehealth accessibility and patient-centred care. One study found that the accessibility of telehealth helped to promote relationship-building with a provider before the patient was required to travel [76]. Patient-centred care, including continuity of care, was reported to be a facilitator in maternity care [53]. Knowing the woman’s story was particularly important in enhancing Aboriginal women’s experience with maternity care [53].

### Quality appraisal

Thirty-nine studies (78%) were assessed using the JBI quality appraisal tool for qualitative design, while sixteen studies (32%) were evaluated using the JBI quality appraisal tool for analytical cross-sectional studies. A supplementary file includes the quality appraisal for all studies (see Supplementary file 3). All qualitative studies (*n* = 39) obtained ethical approval and adequately discussed the congruity between the research methodology, data collection methods, data analysis, and data interpretation. Most studies (*n* = 38) addressed the congruity between the research methodology and objectives. However, there were less common reporting on philosophical perspective (*n* = 15), locating the researcher culturally or theoretically (*n* = 9), and researcher reflexivity (*n* = 6). All cross-sectional studies (*n* = 16) adequately discussed the inclusion criteria, participants, settings, and outcome measures. Confounding variables were the lowest met criteria among studies, with majority of studies (*n* = 12) identifying them; however, only a small number (*n* = 2) stated strategies to deal with them.

## Discussion

To our knowledge, this is the first review to synthesise the evidence on the spatial and aspatial barriers and facilitators that women experience in accessing SRH services in rural healthcare settings in Australia. This review highlighted significant barriers and facilitators from supply and demand access dimensions.

Many of the facilitators to SRH access identified in this review relate to women’s empowerment. Empowerment refers to the ability of women to make choices and exercise agency and decision-making through expanded access to and control over resources and changes to the institutional structures that affect their lives [78, 79]. Women’s agency is crucial to accessing and utilising SRH services [80], and the availability and accessibility of such services can impact their ability to exercise agency. For example, rural women face numerous challenges in exercising agency compared to their urban counterparts due to limited choices from the scarcity of local resources and the need to travel longer distances to access services, as demonstrated in the included studies [46]. In this review, the dimension of availability and accommodation and the ability to reach were examined the most across studies. It included multiple supply (poor local access, inadequate health service availability, limited provider availability) and demand (transportation issues, increased travel) barriers. The geographical disparity not only limits rural women’s autonomy in making decisions regarding their SRH but also worsens existing barriers across other dimensions, such as affordability, as the financial burden of travelling for care further impedes their ability to access essential SRH services. This translates to mean that simply living outside of a major city reduces women’s agency in terms of their sexual and reproductive health; and further exacerbates health inequities experienced in rural communities.

Health literacy can contribute to women’s empowerment and agency by giving them the tools to access, understand, and use health information to participate in shared decision-making, which is essential for enabling patient-centred care [81]. Improving health literacy and better information dissemination were facilitators identified under the dimension of approachability and the ability to perceive, particularly for STI screening and testing, and culturally appropriate health information. For example, one study [54] recommended comprehensive, culturally respectful, and accessible information on puberty and menstruation in the school health curriculum to improve health literacy for adolescents in rural areas.

Patient-centred care was a facilitator identified in the appropriateness and ability to engage dimension. Continuity of carer was identified as a significant factor in access to maternity care, also highlighted in other systematic reviews of access to antenatal care in high-income countries [13]. A person-centred policy, practice, and research approach can contribute to empowerment. Healthcare systems can mitigate gendered disparities in healthcare access and quality by prioritising the perspectives and experiences of women [82, 83]. Overlooking women’s perspectives in examining access can lead to a failure to fully understand their needs, preferences, and experiences within the healthcare system, which may perpetuate gendered disparities and inefficiencies in healthcare delivery. To address this, a collaborative approach that actively incorporates the perspectives of all stakeholders whilst applying a gendered lens is essential in the design and delivery of care for women [82, 83]. Greater emphasis on co-design and active patient involvement in research and service development can lead to more patient-centred healthcare systems that address inequities and better meet the diverse needs of individuals and communities, particularly in the rural context [84]. Co-designed SRH research is currently underway in Australia. For example, co-designed nurse-led models of care to increase rural access to medical abortion and contraception [85].

Despite emphasising patient-centred care, negative provider attitudes pose significant barriers to healthcare access, particularly accessing abortion care in rural areas. A recent scoping review highlighted the relationship between negative attitudes, abortion stigma and quality of care, contributing to the gatekeeping and obstruction of abortion access [86]. Negative provider attitudes and low-quality care were supply barriers identified under the dimension of acceptability and ability to seek. The reluctance of some GPs to refer for abortion services or their denial of care altogether contributed to the challenges women faced in accessing essential reproductive health services. Interventions that address provider attitudes and promote non-judgmental, patient-centred care are essential to ensure that women in rural areas receive comprehensive healthcare. Provider interventions that have been suggested include provider peer support groups, skills building and education to improve patient-provider interactions, strategies for combating negative behaviours, and addressing explicit and implicit biases [86, 87].

In addition to addressing negative provider attitudes, it is crucial to acknowledge the affordability and ability to pay dimension, as the financial barriers further hinder access to essential SRH services for women in rural areas. Some women in the included studies reported a lack of information and difficulties accessing financial incentives for maternity care relocation, such as Patient Assisted Travel Scheme (PATS) [46]. PATS eligibility and payment rates vary between states and territories [88]; however, this information should be more readily available through community education and health services. Limited financial support for patients and providers alike poses significant barriers to the affordability of healthcare in rural areas [46, 63, 68, 74, 75, 89, 90]. The closure of rural services not only shifts costs within health regions but also burdens service users with additional financial responsibilities, such as the indirect costs of travelling further for healthcare [74]. Already, rural and remote residents tend to be of a lower socioeconomic status and more disadvantaged, exacerbating these impacts [91, 92].

Healthcare expenses paid directly by individuals in Australia comprise 17 per cent of the country’s healthcare expenditures [93]. Women, in particular, bear a significant portion of these costs, with their out-of-pocket healthcare spending surpassing that of men [93]. Medicare and the Pharmaceutical Benefits Scheme (PBS) require a substantial overhaul to review rebates and subsidised items for women’s sexual and reproductive care, including rebates for GPs and nursing practitioners [89, 90, 94]. Funded positions for rural SRH GPs can alleviate financial pressures and encourage clinicians to provide comprehensive SRH care [89]. By addressing these financial barriers and implementing supportive policies, the government can ensure that women and providers in rural areas have the resources they need to access and provide quality healthcare.

Beyond the immediate health implications, it is essential to recognise the broader societal benefits associated with improving access to SRH services in rural communities. Addressing the barriers to accessing SRH services in rural areas holds wider societal benefits, including economic and social outcomes [7, 8]. For instance, it is well documented that investing in and improving access to SRH care can lead to substantial cost savings by reducing the burden on healthcare systems associated with preventable or untreated SRH issues [8, 95]. The proposed policy recommendations from this review align with national and international frameworks, such as the National Women’s Health Strategy [30] and Sustainable Development Goals [96], prioritising universal healthcare access and gender equity. By implementing these policy interventions, governments can improve women’s health and well-being in rural areas, and address health inequities.

### Recommendations and policy implications

Considering the critical need to address barriers to SRH access in rural Australia, policymakers must prioritise targeted interventions to redress disparities in SRH service accessibility. First, there is a pressing need for health service redesign and interventions prioritising women’s empowerment and control over their health. Further research across different states, particularly those with significant rural populations (e.g., Western Australia), is essential to capture the diverse needs of these communities and ensure patient-centred care. This includes the prioritisation of Aboriginal and Torres Strait Islander women’s voices in developing culturally safe antenatal care models and addressing barriers to antenatal and birthing care, especially in rural and remote Australia. Further, there needs to be a consistent use of geographic classification systems relevant to policy in rural research to facilitate decision-making from findings [97, 98]. Second, a comprehensive review of Medicare rebates and PBS subsidised items is warranted to enhance affordability and accessibility to SRH care, including the re-introduction of patient rebates for longer in-person and telehealth consultations. This would enable access to care for women in rural and remote areas and with complex health needs, as well as enable patients to access rebates for services and procedures performed by a nurse practitioner. Additionally, fully subsidised, or low out-of-pocket costs for abortion care and access to the full range of contraceptive options in rural areas are crucial. Third, initiatives to enhance healthcare provider education on SRH issues, particularly addressing negative attitudes and biases, are imperative and could start as early as during medical training for students to understand their own biases. Free or government-subsidised rural GP education and training across all sexual, reproductive, and gender-diverse areas can further incentivise their involvement in providing crucial healthcare services to women in rural communities. Finally, investing in digital healthcare infrastructure is crucial for equitable and affordable SRH service access for rural populations. Critical to this is Medicare’s continued recognition of these services.

### Strengths and limitations

A strength of this systematic review is the use of rigorous and robust methods to identify, appraise and synthesise the literature pertaining to the barriers and facilitators to spatial and aspatial access to women’s SRH services in rural Australian healthcare settings. In particular, the comprehensive search and selection process identified a large number of included studies published in the last ten years. This review builds on existing evidence to understand health service accessibility in Australia [1] and aligns with national policy focused on addressing workforce maldistribution, ultimately improving healthcare access [99]. While this review aimed to include all essential SRH services, there were gaps in the available literature that may limit the generalisability of identified barriers and facilitators for services not captured within the included studies. Specifically, this review did not identify any studies examining the barriers and facilitators of access to contraception (including long-acting reversible contraception), gestational diabetes care, early pregnancy assessment services, and menopause services. An additional consideration is the underrepresentation of studies focused on specific population sub-groups and Australian states. Only six studies focussed on Aboriginal and Torres Strait Islander people’s access to care, and most studies were conducted in NSW and Victoria. Lastly, while eligibility for this review restricted inclusion to peer-reviewed studies, the exclusion of grey literature, such as health reports, may have heightened the potential for publication bias. Future research or reviews could examine access more broadly to contribute to understanding barriers and facilitators for the SRH services missed in this review.

## Conclusion

Identifying the barriers and facilitators for women in accessing essential SRH services within the rural context is a necessary step for the development of comprehensive healthcare policies and interventions that address the diverse needs of rural women. Our study supports the need for targeted interventions to redress disparities in SRH service accessibility in rural areas. These include a comprehensive review of Medicare rebates and PBS subsidised items to enhance affordability and accessibility to SRH care, investment in digital healthcare infrastructure, and health service redesign prioritising co-design and a person-centred approach to ensure the service meets the needs of rural women. Further research is required to target the access barriers to SRH services and address the disparities in health service distribution and health inequity in rural Australia.

## Supplementary Information

Supplementary Material 1.

Supplementary Material 2.

Supplementary Material 3.

Supplementary Material 4.

## Data Availability

No datasets were generated or analysed during the current study.

## Abbreviations

AHP: Aboriginal health practitioners
AIHW: Australian Institute of Health and Welfare
ASGC-RA: Australian Statistical Geography Classification–Remoteness Area
ASGS-RA: Australian Statistical Geography Standard–Remoteness Area
ART: Assisted reproductive technology
EC: Emergency contraception
GP: General practitioner
HPV: Human papillomavirus
IVF: In vitro fertilisation
IUD: Intrauterine device
MA: Medical abortion
MMM: Modified Monash Model
MToP: Medical termination of pregnancy
NCSP: National Cervical Screening Program
NSW: New South Wales
NT: Northern Territory
PATS: Patient Assisted Travel Scheme
PBS: Pharmaceutical Benefits Scheme
PMU: Primary maternity units
PN: Practice nurses
POCT: Point-of-care-test
QLD: Queensland
SRH: Sexual and reproductive health
SA: South Australia
STI: Sexually transmitted infection
SToP: Surgical termination of pregnancy
TAS: Tasmania
WA: Western Australia
VIC: Victoria
