# Supplementary material for: Barriers and facilitators to women’s access to sexual and reproductive health services in rural Australia: a systematic review
Source: BMC Health Serv Res. 2024 Oct 11;24:1221. doi: 10.1186/s12913-024-11710-9 (PMC11468210; doi:10.1186/s12913-024-11710-9)
Supplement: Supplementary file 1 — Supplementary Material 1. [file 12913_2024_11710_MOESM1_ESM.docx]

**Supplementary File 2. Database searches**

| **Database** | **Citations retrieved** |
| --- | --- |
| MEDLINE Complete | 600 |
| Embase (Elsevier) | 650 |
| CINAHL (EBSCOhost) | 410 |
| Health Policy Reference Center (EBSCOhost) | 80 |
| Global Health (EBSCOhost) | 239 |
| Health Source: Nursing/Academic Edition (EBSCOhost) | 79 |
| Total | 2058 |
| Duplicate papers | 1034 |
| **Total left to screen** | **1024** |

| **Database: MEDLINE Complete (EBSCOhost)** |
| --- |
| 1. TI "birth control" OR AB" birth control" 2. TI "family planning" OR AB "family planning" 3. TI IUD OR AB IUD 4. TI LARC OR AB LARC 5. TI "sexually transmitted infection*” OR AB "sexually transmitted infection*” 6. TI “STI screen*” OR AB “STI screen*” 7. TI “STI test*” OR AB “STI test*” 8. TI “medical termination” OR AB “medical termination” 9. TI “surgical termination” OR AB “surgical termination” 10. TI “pregnancy termination” OR AB “pregnancy termination” 11. TI “medical abortion” OR AB “medical abortion” 12. TI “surgical abortion” OR AB “surgical abortion” 13. TI “abortion care” OR AB “abortion care” 14. TI “spontaneous abortion” OR AB “spontaneous abortion” 15. TI “obstetric*” OR AB “obstetric*” 16. TI “maternity service*” OR AB “maternity service*” 17. TI “abortion service*” OR AB “abortion service*” 18. TI “fertility service*” OR AB “fertility service*” 19. TI “IVF service*” OR AB “IVF service*” 20. TI “sexual health service*” OR AB “sexual health service*” 21. TI “reproductive service*” OR AB “reproductive service*” 22. TI “pregnancy care” OR AB “pregnancy care” 23. TI “maternity care” OR AB “maternity care” 24. TI “antenatal care” OR AB “antenatal care” 25. TI “perinatal care” OR AB “perinatal care” 26. TI “postnatal care” OR AB “postnatal care” 27. TI “miscarriage care” OR AB “miscarriage care” 28. TI “sexual health” OR AB “sexual health” 29. TI “reproductive health” OR AB “reproductive health” 30. TI “maternal health” OR AB “maternal health” 31. TI “perinatal health” OR AB “perinatal health” 32. TI “postnatal health” OR AB “postnatal health” 33. TI “women* health” OR AB “women* health” 34. MH "contraception"+ 35. MH "intrauterine devices+” 36. MH "reproductive techniques"+ 37. MH "menstrual cycle"+ 38. MH “menstruation disturbances”+ 39. MH “menopause”+ 40. MH “gynecology”+ 41. MH "sexually transmitted diseases"+ 42. MH "women's health services"+ 43. MH "women's health"+ 44. 1-43 /or 45. TI “spatial access” OR AB “spatial access” 46. TI “aspatial access” OR AB “aspatial access” 47. TI “potential access” OR AB “potential access” 48. TI “reali?ed access” OR AB “reali?ed access” 49. TI access OR AB access 50. TI accessibility OR AB accessibility 51. TI proximity OR AB proximity 52. TI travel* OR AB travel* 53. TI distance OR AB distance 54. TI availability OR AB availability 55. TI location* OR AB location* 56. TI distribution OR AB distribution 57. TI provider OR AB provider 58. TI provision OR AB provision 59. TI supply OR AB supply 60. TI demand OR AB demand 61. TI affordability OR AB affordability 62. TI cost OR AB cost 63. TI acceptability OR AB acceptability 64. TI attitudes OR AB attitudes 65. TI experience OR AB experience 66. TI cultural OR AB cultural 67. TI trust OR AB trust 68. TI timeliness OR AB timeliness 69. TI "wait* times" OR AB "wait* times" 70. TI "wait* list*” OR AB "wait* list*” 71. TI accommodation OR AB accommodation 72. TI usability OR AB usability 73. TI adequ* OR AB adequ* 74. TI awareness OR AB awareness 75. TI knowledge OR AB knowledge 76. TI “health literacy” OR AB “health literacy” 77. TI barrier* OR AB barrier* 78. TI obstacle* OR AB obstacle* 79. TI challeng* OR AB challeng* 80. TI enable* OR AB enable* 81. TI facilitat* OR AB facilitat* 82. 45-81/or 83. TI rural* OR AB rural* 84. TI remote* OR AB remote* 85. TI region* OR AB region* 86. 83-85/or 87. TI Australia* OR AB Australia* 88. TI Tasmania* OR AB Tasmania* 89. TI Victoria* OR AB Victoria* 90. TI “New South Wales” OR AB “New South Wales” 91. TI Queensland* OR AB Queensland* 92. TI “Northern Territor*” OR AB “Northern Territor*” 93. TI “Western Australia*” OR AB “Western Australia*” 94. TI “South Australia*” OR AB “South Australia*” 95. MH “Australia”+ 96. 87-95/or 97. 44 AND 82 AND 86 AND 96 98. Limiters - Date of Publication: 20130101-20231231 |

| **Database: EMBASE (Elsevier)** |
| --- |
| 1. ‘birth control’:ab,ti 2. ‘family planning’:ab,ti 3. IUD:ab,ti 4. LARC:ab,ti 5. ‘sexually transmitted infection*’:ab,ti 6. ‘STI screen*’:ab,ti 7. ‘STI test*’:ab,ti 8. ‘medical termination’:ab,ti 9. ‘surgical termination’:ab,ti 10. ‘pregnancy termination’:ab,ti 11. ‘medical abortion’:ab,ti 12. ‘surgical abortion’:ab,ti 13. ‘abortion care’:ab,ti 14. ‘spontaneous abortion’:ab,ti 15. ‘obstetric*’:ab,ti 16. ‘maternity service*’:ab,ti 17. ‘abortion service*’:ab,ti 18. ‘fertility service*’:ab,ti 19. ‘IVF service*’:ab,ti 20. ‘sexual health service*’:ab,ti 21. ‘reproductive service*’:ab,ti 22. ‘pregnancy care’:ab,ti 23. ‘antenatal care’:ab,ti 24. ‘perinatal care’:ab,ti 25. ‘postnatal care’:ab,ti 26. ‘miscarriage care’:ab,ti 27. ‘sexual health’:ab,ti 28. ‘reproductive health’:ab,ti 29. ‘maternal health’:ab,ti 30. ‘perinatal health’:ab,ti 31. ‘postnatal health’:ab,ti 32. ‘women* health’:ab,ti 33. ‘contraception’/de 34. ‘female contraceptive device’/de 35. ‘infertility therapy’/de 36. ‘menstrual cycle’/de 37. ‘gynecologic disease’/de 38. ‘menopause’/de 39. ‘gynecologic surgery’/de 40. ‘sexually transmitted disease’/de 41. ‘maternal care’/de 42. ‘women's health’/de 43. 1-42 /or 44. ‘spatial access’:ab,ti 45. ‘aspatial access’:ab,ti 46. ‘potential access’:ab,ti 47. ‘reali?ed access’:ab,ti 48. access:ab,ti 49. accessibility:ab,ti 50. proximity:ab,ti 51. travel*:ab,ti 52. distance:ab,ti 53. availability:ab,ti 54. location*:ab,ti 55. distribution:ab,ti 56. provider:ab,ti 57. provision:ab,ti 58. supply:ab,ti 59. demand:ab,ti 60. affordability:ab,ti 61. cost:ab,ti 62. acceptability:ab,ti 63. attitudes:ab,ti 64. experience:ab,ti 65. cultural:ab,ti 66. trust:ab,ti 67. timeliness:ab,ti 68. ‘wait* times’:ab,ti 69. ‘wait* list*’:ab,ti 70. accommodation:ab,ti 71. usability:ab,ti 72. adequ*:ab,ti 73. awareness:ab,ti 74. knowledge:ab,ti 75. ‘health literacy’:ab,ti 76. barrier*:ab,ti 77. obstacle*:ab,ti 78. challeng*:ab,ti 79. enable*:ab,ti 80. facilitat*:ab,ti 81. 45-80/or 82. rural*:ab,ti 83. remote*:ab,ti 84. region*:ab,ti 85. ‘rural population’/de 86. 82-85/or 87. Australia*:ab,ti 88. Tasmania*:ab,ti 89. Victoria*:ab,ti 90. ‘New South Wales’:ab,ti 91. Queensland*:ab,ti 92. ‘Northern Territor*’:ab,ti 93. ‘Western Australia*’:ab,ti 94. ‘South Australia*’:ab,ti 95. ‘Australia’/de 96. 87-95/or 97. 43 AND 81 AND 86 AND 96 98. Limiters - Date of Publication: 20130101-20231231 |

| **Database: CINAHL Complete (EBSCOhost)** |
| --- |
| 1. TI "birth control" OR AB" birth control" 2. TI "family planning" OR AB "family planning" 3. TI IUD OR AB IUD 4. TI “intrauterine device*” OR AB “intrauterine device*” 5. TI LARC OR AB LARC 6. TI "sexually transmitted infection*” OR AB "sexually transmitted infection*” 7. TI “STI screen*” OR AB “STI screen*” 8. TI “STI test*” OR AB “STI test*” 9. TI “medical termination” OR AB “medical termination” 10. TI “surgical termination” OR AB “surgical termination” 11. TI “pregnancy termination” OR AB “pregnancy termination” 12. TI “medical abortion” OR AB “medical abortion” 13. TI “surgical abortion” OR AB “surgical abortion” 14. TI “abortion care” OR AB “abortion care” 15. TI “spontaneous abortion” OR AB “spontaneous abortion” 16. TI “obstetric*” OR AB “obstetric*” 17. TI “maternity service*” OR AB “maternity service*” 18. TI “abortion service*” OR AB “abortion service*” 19. TI “fertility service*” OR AB “fertility service*” 20. TI “IVF service*” OR AB “IVF service*” 21. TI “sexual health service*” OR AB “sexual health service*” 22. TI “reproductive service*” OR AB “reproductive service*” 23. TI “pregnancy care” OR AB “pregnancy care” 24. TI “maternity care” OR AB “maternity care” 25. TI “antenatal care” OR AB “antenatal care” 26. TI “perinatal care” OR AB “perinatal care” 27. TI “postnatal care” OR AB “postnatal care” 28. TI “miscarriage care” OR AB “miscarriage care” 29. TI “sexual health” OR AB “sexual health” 30. TI “reproductive health” OR AB “reproductive health” 31. TI “maternal health” OR AB “maternal health” 32. TI “perinatal health” OR AB “perinatal health” 33. TI “postnatal health” OR AB “postnatal health” 34. TI “women* health” OR AB “women* health” 35. MH "contraception"+ 36. MH "reproduction techniques"+ 37. MH "menstrual cycle"+ 38. MH “menstruation disorders”+ 39. MH “menopause”+ 40. MH “Surgery, Ob-Gyn”+ 41. MH "sexually transmitted diseases"+ 42. 1-41 /or 43. TI “spatial access” OR AB “spatial access” 44. TI “aspatial access” OR AB “aspatial access” 45. TI “potential access” OR AB “potential access” 46. TI “reali?ed access” OR AB “reali?ed access” 47. TI access OR AB access 48. TI accessibility OR AB accessibility 49. TI proximity OR AB proximity 50. TI travel* OR AB travel* 51. TI distance OR AB distance 52. TI availability OR AB availability 53. TI location* OR AB location* 54. TI distribution OR AB distribution 55. TI provider OR AB provider 56. TI provision OR AB provision 57. TI supply OR AB supply 58. TI demand OR AB demand 59. TI affordability OR AB affordability 60. TI cost OR AB cost 61. TI acceptability OR AB acceptability 62. TI attitudes OR AB attitudes 63. TI experience OR AB experience 64. TI cultural OR AB cultural 65. TI trust OR AB trust 66. TI timeliness OR AB timeliness 67. TI "wait* times" OR AB "wait* times" 68. TI "wait* list*” OR AB "wait* list*” 69. TI accommodation OR AB accommodation 70. TI usability OR AB usability 71. TI adequ* OR AB adequ* 72. TI awareness OR AB awareness 73. TI knowledge OR AB knowledge 74. TI “health literacy” OR AB “health literacy” 75. TI barrier* OR AB barrier* 76. TI obstacle* OR AB obstacle* 77. TI challeng* OR AB challeng* 78. TI enable* OR AB enable* 79. TI facilitat* OR AB facilitat* 80. 43-79/or 81. TI rural* OR AB rural* 82. TI remote* OR AB remote* 83. TI region* OR AB region* 84. 81-83/or 85. TI Australia* OR AB Australia* 86. TI Tasmania* OR AB Tasmania* 87. TI Victoria* OR AB Victoria* 88. TI “New South Wales” OR AB “New South Wales” 89. TI Queensland* OR AB Queensland* 90. TI “Northern Territor*” OR AB “Northern Territor*” 91. TI “Western Australia*” OR AB “Western Australia*” 92. TI “South Australia*” OR AB “South Australia*” 93. MH “Australia”+ 94. 85-93/or 95. 42 AND 80 AND 84 AND 94 96. Limiters - Date of Publication: 20130101-20231231 |

| **Database: Health Policy Reference Center (EBSCOhost)** |
| --- |
| 1. TI "birth control" OR AB" birth control" 2. TI "family planning" OR AB "family planning" 3. TI IUD OR AB IUD 4. TI “intrauterine device*” OR AB “intrauterine device*” 5. TI LARC OR AB LARC 6. TI "sexually transmitted infection*” OR AB "sexually transmitted infection*” 7. TI “STI screen*” OR AB “STI screen*” 8. TI “STI test*” OR AB “STI test*” 9. TI “medical termination” OR AB “medical termination” 10. TI “surgical termination” OR AB “surgical termination” 11. TI “pregnancy termination” OR AB “pregnancy termination” 12. TI “medical abortion” OR AB “medical abortion” 13. TI “surgical abortion” OR AB “surgical abortion” 14. TI “abortion care” OR AB “abortion care” 15. TI “spontaneous abortion” OR AB “spontaneous abortion” 16. TI “obstetric*” OR AB “obstetric*” 17. TI “maternity service*” OR AB “maternity service*” 18. TI “abortion service*” OR AB “abortion service*” 19. TI “fertility service*” OR AB “fertility service*” 20. TI “IVF service*” OR AB “IVF service*” 21. TI “sexual health service*” OR AB “sexual health service*” 22. TI “reproductive service*” OR AB “reproductive service*” 23. TI “pregnancy care” OR AB “pregnancy care” 24. TI “maternity care” OR AB “maternity care” 25. TI “antenatal care” OR AB “antenatal care” 26. TI “perinatal care” OR AB “perinatal care” 27. TI “postnatal care” OR AB “postnatal care” 28. TI “miscarriage care” OR AB “miscarriage care” 29. TI “sexual health” OR AB “sexual health” 30. TI “reproductive health” OR AB “reproductive health” 31. TI “maternal health” OR AB “maternal health” 32. TI “perinatal health” OR AB “perinatal health” 33. TI “postnatal health” OR AB “postnatal health” 34. TI “women* health” OR AB “women* health” 35. DE "contraception" 36. DE "reproductive health services" 37. DE "menstrual cycle" 38. DE “menstruation disorders” 39. DE “menopause” 40. DE “Obstetrics” 41. DE “Gynecology” 42. DE “Women’s health services” 43. DE "sexually transmitted diseases" 44. DE “Abortion” 45. 1-44 /or 46. TI “spatial access” OR AB “spatial access” 47. TI “potential access” OR AB “potential access” 48. TI “reali?ed access” OR AB “reali?ed access” 49. TI access OR AB access 50. TI accessibility OR AB accessibility 51. TI proximity OR AB proximity 52. TI travel* OR AB travel* 53. TI distance OR AB distance 54. TI availability OR AB availability 55. TI location* OR AB location* 56. TI distribution OR AB distribution 57. TI provider OR AB provider 58. TI provision OR AB provision 59. TI supply OR AB supply 60. TI demand OR AB demand 61. TI affordability OR AB affordability 62. TI cost OR AB cost 63. TI acceptability OR AB acceptability 64. TI attitudes OR AB attitudes 65. TI experience OR AB experience 66. TI cultural OR AB cultural 67. TI trust OR AB trust 68. TI timeliness OR AB timeliness 69. TI "wait* times" OR AB "wait* times" 70. TI "wait* list*” OR AB "wait* list*” 71. TI accommodation OR AB accommodation 72. TI usability OR AB usability 73. TI adequ* OR AB adequ* 74. TI awareness OR AB awareness 75. TI knowledge OR AB knowledge 76. TI “health literacy” OR AB “health literacy” 77. TI barrier* OR AB barrier* 78. TI obstacle* OR AB obstacle* 79. TI challeng* OR AB challeng* 80. TI enable* OR AB enable* 81. TI facilitat* OR AB facilitat* 82. 46-81/or 83. TI rural* OR AB rural* 84. TI remote* OR AB remote* 85. TI region* OR AB region* 86. 83-85/or 87. TI Australia* OR AB Australia* 88. TI Tasmania* OR AB Tasmania* 89. TI Victoria* OR AB Victoria* 90. TI “New South Wales” OR AB “New South Wales” 91. TI Queensland* OR AB Queensland* 92. TI “Northern Territor*” OR AB “Northern Territor*” 93. TI “Western Australia*” OR AB “Western Australia*” 94. TI “South Australia*” OR AB “South Australia*” 95. 87-94/or 96. 45 AND 82 AND 86 AND 95 97. Limiters - Date of Publication: 20130101-20231231 |

| **Database: Global Health (EBSCOhost)** |
| --- |
| - 1. TI "birth control" OR AB" birth control"   2. TI "family planning" OR AB "family planning"   3. TI IUD OR AB IUD   4. TI “intrauterine device*” OR AB “intrauterine device*”   5. TI LARC OR AB LARC   6. TI "sexually transmitted infection*” OR AB "sexually transmitted infection*”   7. TI “STI screen*” OR AB “STI screen*”   8. TI “STI test*” OR AB “STI test*”   9. TI “medical termination” OR AB “medical termination”   10. TI “surgical termination” OR AB “surgical termination”   11. TI “pregnancy termination” OR AB “pregnancy termination”   12. TI “medical abortion” OR AB “medical abortion”   13. TI “surgical abortion” OR AB “surgical abortion”   14. TI “abortion care” OR AB “abortion care”   15. TI “spontaneous abortion” OR AB “spontaneous abortion”   16. TI “obstetric*” OR AB “obstetric*”   17. TI “maternity service*” OR AB “maternity service*”   18. TI “abortion service*” OR AB “abortion service*”   19. TI “fertility service*” OR AB “fertility service*”   20. TI “IVF service*” OR AB “IVF service*”   21. TI “sexual health service*” OR AB “sexual health service*”   22. TI “reproductive service*” OR AB “reproductive service*”   23. TI “pregnancy care” OR AB “pregnancy care”   24. TI “maternity care” OR AB “maternity care”   25. TI “antenatal care” OR AB “antenatal care”   26. TI “perinatal care” OR AB “perinatal care”   27. TI “postnatal care” OR AB “postnatal care”   28. TI “miscarriage care” OR AB “miscarriage care”   29. TI “sexual health” OR AB “sexual health”   30. TI “reproductive health” OR AB “reproductive health”   31. TI “maternal health” OR AB “maternal health”   32. TI “perinatal health” OR AB “perinatal health”   33. TI “postnatal health” OR AB “postnatal health”   34. TI “women* health” OR AB “women* health”   35. DE "contraceptives"+   36. DE "menstruation "+   37. DE “reproductive disorders”+   38. DE “menopause”+   39. DE “Obstetrics”+   40. DE “Gynaecology”+   41. DE "sexually transmitted diseases" +   42. DE “Abortion”+   43. 1-44 /or   44. TI “spatial access” OR AB “spatial access”   45. TI “potential access” OR AB “potential access”   46. TI “reali?ed access” OR AB “reali?ed access”   47. TI access OR AB access   48. TI accessibility OR AB accessibility   49. TI proximity OR AB proximity   50. TI travel* OR AB travel*   51. TI distance OR AB distance   52. TI availability OR AB availability   53. TI location* OR AB location*   54. TI distribution OR AB distribution   55. TI provider OR AB provider   56. TI provision OR AB provision   57. TI supply OR AB supply   58. TI demand OR AB demand   59. TI affordability OR AB affordability   60. TI cost OR AB cost   61. TI acceptability OR AB acceptability   62. TI attitudes OR AB attitudes   63. TI experience OR AB experience   64. TI cultural OR AB cultural   65. TI trust OR AB trust   66. TI timeliness OR AB timeliness   67. TI "wait* times" OR AB "wait* times"   68. TI "wait* list*” OR AB "wait* list*”   69. TI accommodation OR AB accommodation   70. TI usability OR AB usability   71. TI adequ* OR AB adequ*   72. TI awareness OR AB awareness   73. TI knowledge OR AB knowledge   74. TI “health literacy” OR AB “health literacy”   75. TI barrier* OR AB barrier*   76. TI obstacle* OR AB obstacle*   77. TI challeng* OR AB challeng*   78. TI enable* OR AB enable*   79. TI facilitat* OR AB facilitat*   80. 45-81/or   81. TI rural* OR AB rural*   82. TI remote* OR AB remote*   83. TI region* OR AB region*   84. 83-85/or   85. TI Australia* OR AB Australia*   86. TI Tasmania* OR AB Tasmania*   87. TI Victoria* OR AB Victoria*   88. TI “New South Wales” OR AB “New South Wales”   89. TI Queensland* OR AB Queensland*   90. TI “Northern Territor*” OR AB “Northern Territor*”   91. TI “Western Australia*” OR AB “Western Australia*”   92. TI “South Australia*” OR AB “South Australia*”   93. 86-94/or   94. 45 AND 82 AND 86 AND 95   95. Limiters - Date of Publication: 20130101-20231231 |

| **Database: Health Source: Nursing/Academic Edition (EBSCOhost)** |
| --- |
| 1. TI "birth control" OR AB" birth control" 2. TI "family planning" OR AB "family planning" 3. TI IUD OR AB IUD 4. TI “intrauterine device*” OR AB “intrauterine device*” 5. TI LARC OR AB LARC 6. TI "sexually transmitted infection*” OR AB "sexually transmitted infection*” 7. TI “STI screen*” OR AB “STI screen*” 8. TI “STI test*” OR AB “STI test*” 9. TI “medical termination” OR AB “medical termination” 10. TI “surgical termination” OR AB “surgical termination” 11. TI “pregnancy termination” OR AB “pregnancy termination” 12. TI “medical abortion” OR AB “medical abortion” 13. TI “surgical abortion” OR AB “surgical abortion” 14. TI “abortion care” OR AB “abortion care” 15. TI “spontaneous abortion” OR AB “spontaneous abortion” 16. TI “obstetric*” OR AB “obstetric*” 17. TI “maternity service*” OR AB “maternity service*” 18. TI “abortion service*” OR AB “abortion service*” 19. TI “fertility service*” OR AB “fertility service*” 20. TI “IVF service*” OR AB “IVF service*” 21. TI “sexual health service*” OR AB “sexual health service*” 22. TI “reproductive service*” OR AB “reproductive service*” 23. TI “pregnancy care” OR AB “pregnancy care” 24. TI “maternity care” OR AB “maternity care” 25. TI “antenatal care” OR AB “antenatal care” 26. TI “perinatal care” OR AB “perinatal care” 27. TI “postnatal care” OR AB “postnatal care” 28. TI “miscarriage care” OR AB “miscarriage care” 29. TI “sexual health” OR AB “sexual health” 30. TI “reproductive health” OR AB “reproductive health” 31. TI “maternal health” OR AB “maternal health” 32. TI “perinatal health” OR AB “perinatal health” 33. TI “postnatal health” OR AB “postnatal health” 34. TI “women* health” OR AB “women* health” 35. DE "contraception" 36. DE "reproductive health services" 37. DE "menstrual cycle" 38. DE “menstruation disorders” 39. DE “menopause” 40. DE “Obstetrics” 41. DE “Gynecology” 42. DE “Women’s health services” 43. DE "sexually transmitted diseases" 44. DE “Abortion” 45. 1-44 /or 46. TI “spatial access” OR AB “spatial access” 47. TI “potential access” OR AB “potential access” 48. TI “reali?ed access” OR AB “reali?ed access” 49. TI access OR AB access 50. TI accessibility OR AB accessibility 51. TI proximity OR AB proximity 52. TI travel* OR AB travel* 53. TI distance OR AB distance 54. TI availability OR AB availability 55. TI location* OR AB location* 56. TI distribution OR AB distribution 57. TI provider OR AB provider 58. TI provision OR AB provision 59. TI supply OR AB supply 60. TI demand OR AB demand 61. TI affordability OR AB affordability 62. TI cost OR AB cost 63. TI acceptability OR AB acceptability 64. TI attitudes OR AB attitudes 65. TI experience OR AB experience 66. TI cultural OR AB cultural 67. TI trust OR AB trust 68. TI timeliness OR AB timeliness 69. TI "wait* times" OR AB "wait* times" 70. TI "wait* list*” OR AB "wait* list*” 71. TI accommodation OR AB accommodation 72. TI usability OR AB usability 73. TI adequ* OR AB adequ* 74. TI awareness OR AB awareness 75. TI knowledge OR AB knowledge 76. TI “health literacy” OR AB “health literacy” 77. TI barrier* OR AB barrier* 78. TI obstacle* OR AB obstacle* 79. TI challeng* OR AB challeng* 80. TI enable* OR AB enable* 81. TI facilitat* OR AB facilitat* 82. 46-81/or 83. TI rural* OR AB rural* 84. TI remote* OR AB remote* 85. TI region* OR AB region* 86. 83-85/or 87. TI Australia* OR AB Australia* 88. TI Tasmania* OR AB Tasmania* 89. TI Victoria* OR AB Victoria* 90. TI “New South Wales” OR AB “New South Wales” 91. TI Queensland* OR AB Queensland* 92. TI “Northern Territor*” OR AB “Northern Territor*” 93. TI “Western Australia*” OR AB “Western Australia*” 94. TI “South Australia*” OR AB “South Australia*” 95. 87-94/or 96. 45 AND 82 AND 86 AND 95 97. Limiters - Date of Publication: 20130101-20231231 |
