# Supplementary material for: Barriers and facilitators to women’s access to sexual and reproductive health services in rural Australia: a systematic review
Source: BMC Health Serv Res. 2024 Oct 11;24:1221. doi: 10.1186/s12913-024-11710-9 (PMC11468210; doi:10.1186/s12913-024-11710-9)
Supplement: Supplementary file 2 — Supplementary Material 2. [file 12913_2024_11710_MOESM2_ESM.docx]

**Supplementary File 3. Quality Appraisal**

| **JBI Quality Appraisal for Qualitative Studies** | | | | | | | | | | |
| --- | --- | --- | --- | --- | --- | --- | --- | --- | --- | --- |
| **First author (year)** | **1. Is there congruity between the stated philosophical perspective and the research methodology?** | **2. Is there congruity between the research methodology and the research question or objectives?** | **3. Is there congruity between the research methodology and the methods used to collect data?** | **4. Is there congruity between the research methodology and the representation and analysis of data?** | **5. Is there congruity between the research methodology and the interpretation of results?** | **6. Is there a statement locating the researcher culturally or theoretically?** | **7. Is the influence of the researcher on the research, and vice- versa, addressed?** | **8. Are participants, and their voices, adequately represented?** | **9. Is the research ethical according to current criteria or, for recent studies, and is there evidence of ethical approval by an appropriate body?** | **10. Do the conclusions drawn in the research report flow from the analysis, or interpretation, of the data?** |
| Bar-Zeev (2014) | U | Y | Y | Y | Y | N | N | Y | Y | Y |
| Brown (2016) | Y | Y | Y | Y | Y | Y | Y | Y | Y | Y |
| Campbell (2014) | U | Y | Y | Y | Y | N | N | Y | Y | Y |
| Cashman (2021) | U | Y | Y | Y | Y | Y | N | Y | Y | Y |
| Christie (2023) | U | U | Y | Y | Y | N | N | Y | Y | Y |
| Dawson (2017) | Y | Y | Y | Y | Y | N | N | Y | Y | Y |
| De Moel-Mandel (2020) | U | Y | Y | Y | Y | N | N | Y | Y | Y |
| Doran (2014) | Y | Y | Y | Y | Y | N | N | Y | Y | Y |
| Doran (2016) | U | Y | Y | Y | Y | N | N | Y | Y | Y |
| Foo (2021) | Y | Y | Y | Y | Y | Y | N | Y | Y | Y |
| Grant (2019) | U | Y | Y | Y | Y | N | N | Y | Y | Y |
| Hoang (2013) | U | Y | Y | Y | Y | N | N | Y | Y | Y |
| Hoang (2014) | U | Y | Y | Y | Y | N | N | Y | Y | Y |
| Hulme-Chambers (2018) | U | Y | Y | Y | Y | Y | Y | Y | Y | Y |
| Hulme-Chambers (2018) | U | Y | Y | Y | Y | N | N | Y | Y | Y |
| Ireland (2020) | Y | Y | Y | Y | Y | N | N | Y | Y | Y |
| Josif (2014) | Y | Y | Y | Y | Y | N | N | Y | Y | Y |
| Keough (2019) | U | Y | Y | Y | Y | N | N | Y | Y | Y |
| Kruss (2014) | Y | Y | Y | Y | Y | N | N | Y | Y | Y |
| Lafferty (2021) | U | Y | Y | Y | Y | N | N | Y | Y | Y |
| Lansbury (2023) | Y | Y | Y | Y | Y | Y | Y | Y | Y | Y |
| Longman (2017) | U | Y | Y | Y | Y | N | N | N | Y | Y |
| Lorch (2015) | U | Y | Y | Y | Y | N | N | Y | Y | Y |
| Makleff (2023) | Y | Y | Y | Y | Y | N | N | Y | Y | Y |
| Malatzky (2022) | U | Y | Y | Y | Y | Y | Y | Y | Y | Y |
| Munns (2021) | U | Y | Y | Y | Y | N | Y | Y | Y | Y |
| Noonan (2022) | U | Y | Y | Y | Y | N | N | Y | Y | Y |
| Noonan (2023) | U | Y | Y | Y | Y | Y | N | Y | Y | Y |
| Roxburgh (2021) | U | Y | Y | Y | Y | N | N | Y | Y | Y |
| Rumbold 2015 | U | Y | Y | Y | Y | N | N | Y | Y | Y |
| Russell 2021 | Y | Y | Y | Y | Y | Y | Y | Y | Y | Y |
| Sassano 2023 | U | Y | Y | Y | Y | N | N | Y | Y | Y |
| Seear 2021 | Y | Y | Y | Y | Y | N | N | Y | Y | Y |
| Shackleton 2023 | Y | Y | Y | Y | Y | N | N | Y | Y | Y |
| Sivertsen 2021 | Y | Y | Y | Y | Y | N | N | Y | Y | Y |
| Telford 2022 | U | Y | Y | Y | Y | N | N | U | Y | Y |
| Wagg 2020 | Y | Y | Y | Y | Y | N | N | Y | Y | Y |
| Wong Shee 2021 | Y | Y | Y | Y | Y | Y | N | Y | Y | Y |
| Zadoroznyj 2013 | U | Y | Y | Y | Y | N | N | Y | Y | Y |

KEY: Y=Yes; N=No; U=Unclear; NA= Not Applicable

| **JBI Quality Appraisal for Cross-Sectional Studies** | | | | | | | | |
| --- | --- | --- | --- | --- | --- | --- | --- | --- |
| **First author (year)** | **1. Were the criteria for inclusion in the sample clearly defined?** | **2. Were the study subjects and the setting described in detail?** | **3. Was the exposure measured in a valid and reliable way?** | **4. Were objective, standard criteria used for measurement of the condition?** | **5. Were confounding factors identified?** | **6. Were strategies to deal with confounding factors stated?** | **7. Were the outcomes measured in a valid and reliable way?** | **8. Was appropriate statistical analysis used?** |
| Arnold (2021) | Y | Y | Y | Y | Y | N | Y | Y |
| Doig (2021) | Y | Y | Y | Y | Y | N | Y | Y |
| Dutton (2020) | Y | Y | Y | Y | Y | N | Y | Y |
| Gosbell (2023) | Y | Y | Y | Y | Y | N | Y | N |
| Gudka (2014) | Y | y | Y | Y | Y | N | Y | N |
| Hennegan (2014) | Y | Y | Y | Y | Y | Y | Y | Y |
| Hoang (2013) | Y | Y | Y | Y | Y | N | Y | Y |
| Hoang (2014) | Y | Y | Y | Y | Y | N | Y | Y |
| Keough (2019) | Y | Y | Y | Y | Y | N | Y | Y |
| Kruske 2016 | Y | Y | Y | Y | N | N | Y | Y |
| Moel-Mandel (2019) | Y | Y | Y | Y | Y | N | Y | Y |
| Roxburgh (2021) | Y | Y | Y | Y | Y | N | Y | Y |
| Rolfe (2017) | Y | Y | Y | Y | Y | Y | Y | Y |
| Subasinghe 2021 | Y | Y | Y | Y | N | N | Y | Y |
| Sweet 2015 | Y | Y | Y | Y | N | N | Y | Y |
| Telford 2022 | Y | Y | Y | Y | N | N | Y | Y |

KEY: Y=Yes; N=No; U=Unclear; NA= Not Applicable

| **JBI Quality Appraisal for Cohort Studies** | | | | | | | | | | | |
| --- | --- | --- | --- | --- | --- | --- | --- | --- | --- | --- | --- |
| **First author (year)** | **1. Were the two groups similar and recruited from the same population?** | **2. Were the exposures measured similarly to assign people to both exposed and unexposed groups?** | **3. Was the exposure measured in a valid and reliable way?** | **4. Were confounding factors identified?** | **5. Were strategies to deal with confounding factors stated?** | **6. Were the groups/ participants free of the outcome at the start of the study (or at the moment of exposure)?** | **7. Were the outcomes measured in a valid and reliable way?** | **8.Was the follow up time reported and sufficient to be long enough for outcomes to occur?** | **9. Was follow up complete, and if not, were the reasons to loss to follow up described and explored?** | **10. Were strategies to address incomplete follow up utilized?** | **11. Was appropriate statistical analysis used?** |
| Bar-Zeev (2014) | Y | Y | Y | N | N | Y | N | Y | NA | NA | Y |

KEY: Y=Yes; N=No; U=Unclear; NA= Not Applicable
