# Supplementary material for: Barriers and facilitators to women’s access to sexual and reproductive health services in rural Australia: a systematic review
Source: BMC Health Serv Res. 2024 Oct 11;24:1221. doi: 10.1186/s12913-024-11710-9 (PMC11468210; doi:10.1186/s12913-024-11710-9)
Supplement: Supplementary file 4 — Supplementary Material 4. [file 12913_2024_11710_MOESM4_ESM.docx]

**Supplementary File 4. Geographic classification systems used to define rurality or remoteness areas**

| **Classification systems** | **No of Studies** |
| --- | --- |
| **ASGS or ASGC Remoteness Areas** | |
| Inner Regional Australia | *n*=8 |
| Outer Regional Australia | *n*=10 |
| Remote Australia | *n*=12 |
| Very Remote Australia | *n*=12 |
| **Modified Monash Model** | |
| Large Rural Towns | *n*=1 |
| Medium Rural Towns | *n*=1 |
| Small Rural Towns | *n*=1 |
| **Classification undefined** |  |
| Regional | *n*=18 |
| Outer Regional | *n*=1 |
| Rural | *n*=12 |
| Remote | *n*=1 |

Key: ASGC-RA: Australian Statistical Geography Classification–Remoteness Area; ASGS-RA: Australian Statistical Geography Standard–Remoteness Area
